# Analyzing the User Behavior toward Electronic Commerce Stimuli

**DOI:** 10.3389/fnbeh.2016.00224

**Published:** 2016-11-30

**Authors:** Carlota Lorenzo-Romero, María-del-Carmen Alarcón-del-Amo, Miguel-Ángel Gómez-Borja

**Affiliations:** ^1^Business Department, University of Castilla-La ManchaAlbacete, Spain; ^2^Business Department, Universidad de AlcaláAlcalá de Henares, Spain

**Keywords:** verbal (utilitarian) and non-verbal (hedonic) computer stimuli, webmosphere tools, Stimulus-Organism-Response model, shopping human behavior, computer experimental design

## Abstract

Based on the Stimulus-Organism-Response paradigm this research analyzes the main differences between the effects of two types of web technologies: Verbal web technology (i.e., navigational structure as utilitarian stimulus) versus non-verbal web technology (music and presentation of products as hedonic stimuli). Specific webmosphere stimuli have not been examined yet as separate variables and their impact on internal and behavioral responses seems unknown. Therefore, the objective of this research consists in analyzing the impact of these web technologies –which constitute the web atmosphere or webmosphere of a website– on shopping human behavior (i.e., users’ internal states -affective, cognitive, and satisfaction- and behavioral responses – approach responses, and real shopping outcomes-) within the retail online store created by computer, taking into account some mediator variables (i.e., involvement, atmospheric responsiveness, and perceived risk). A 2 (“free” versus “hierarchical” navigational structure) × 2 (“on” versus “off” music) × 2 (“moving” versus “static” images) between-subjects computer experimental design is used to test empirically this research. In addition, an integrated methodology was developed allowing the simulation, tracking and recording of virtual user behavior within an online shopping environment. As main conclusion, this study suggests that the positive responses of online consumers might increase when they are allowed to freely navigate the online stores and their experience is enriched by animate gifts and music background. The effect caused by mediator variables modifies relatively the final shopping human behavior.

## Introduction

Kotler (1973) defines atmospherics as “the conscious designing of space to create certain buyer effects, specifically, the designing of buying environments to produce specific emotional effects in the buyer that enhance purchase probability.” Given the extended increasing of online stores, some authors are focusing their research on studying an “extended” term: Web atmospheric, also called webmosphere by Childers et al. (2001), or virtual store atmosphere by Vrechopoulos et al. (2000), or online store environment by Manganari et al. (2009). This concept is defined by Dailey (2004) as “the conscious designing of web environments to create positive effects (e.g., positive affect, positive cognitions, etc.) in users in order to obtain more positive responses (e.g., site revisiting or browsing). When marketers design web interfaces in order to entice consumers, they are utilizing web atmospherics.”

Childers et al. (2001) explain that the use the use of video, images, humor, color, music, and other interactive aspects can define and hedonic experience and help to create a more enjoyable environment. On the contrary, a technology oriented perspective that focus on shopping as a cold information systems, with no enjoyable environments, is probable to be decreased, specially for products with hedonic attributes (Hirschman and Holbrook, 1982; Childers et al., 2001; Solaymani et al., 2012).

Most authors use the Stimulus-Organism-Response (S-O-R) paradigm proposed by Mehrabian and Russell (1974) to explain the influence of web atmosphere on consumers. These authors suggest that external stimulus (like web atmospheric cues) affect consumers’ internal states and, in turn, they have an effect on behavioral responses. In the online shopping environment, some researchers use actual stimuli (Wang et al., 2010; Animesh et al., 2011; Kim and Lennon, 2013), and others use customer assessments of the stimuli to denote the stimulus segment of the model (Nath, 2009; Koo and Ju, 2010; Manganari et al., 2011). Eroglu et al. (2001, 2003) proposed two types of webmospheric cues which named high and low task relevant cues. High task-relevant cues comprise verbal or pictorial contents directly associated with the shopping goal. The purpose of these verbal or pictorial descriptions (e.g., product information, price, and delivery and return policies) is to assist online consumers to reach their shopping goals. Low task relevant cues, on the other hand, are peripheral contents (e.g., color, background patterns, and images) not directly related to the shopping goals. Moreover, the organism (i.e., user’s internal states of S-O-R model) is composed by affective, cognitive and satisfaction variables (Oliver, 1997; Bigné and Andreu, 2004). In addition, Eroglu et al. (2001, 2003) suggest that the internal states of the organism include affect and cognition and that satisfaction is one of the outcomes of behavior. And, finally, the response (i.e., behavioral responses of S-O-R model) refer to website patronage intention (Jeong et al., 2009), purchase intention (Hsu et al., 2011; Liu et al., 2016), and intention to use and buy (Huang, 2013).

Many studies do not examine specific webmosphere stimuli separately and their impact on internal and behavioral responses. Normally, these studies analyze global stimuli such as high and low task relevant cues (Eroglu et al., 2001, 2003) and their impact on consumer. Other authors use only one web stimulus like navigation structure (Dailey, 2004) including some aspects referred to web navigation. Other studies analyze specific stimuli attending to other online marketing variables such as price, service quality, social variables, etc. (Chang et al., 2011; Nesset et al., 2011), not focusing the research toward web design exclusively. Moreover, some previous research analyzes behavioral responses such as purchase intention against real shopping (although through simulation). Previous research does not examine the relationship between three mediator variables. Some authors analyze perceived risk or trust (Kim, 2012), or product involvement (Keng et al., 2012), or atmospheric responsiveness (Eroglu et al., 2001). Nevertheless, any study analyzes the possible relationship and impact of three constructs on internal and behavioral online buyer responses.

Thus, the main objective of this study is to establish the main different effects of two webmosphere verbal communication stimulus (i.e., navigation structure) and non-verbal communication stimuli (i.e., music and presentation of products), on internal (i.e., satisfaction, cognitive and affective) and behavioral consumer responses (i.e., approach responses and real shopping outcomes) within a virtual apparel shopping environment. Moreover, three mediator variables (i.e., involvement, atmospheric responsiveness, and perceived risk) are considered in the study in order to analyze the impact of them on the relationships between webmospheric cues and user responses.

Specifically, affective state refers to emotions that user feels toward different stimuli (Eroglu et al., 2001, 2003). Cognitive state is the thought processes and state of mind related to the acquisition, processing, retention, and retrieval of information (Eroglu et al., 2001). Satisfaction is understood as a global evaluation or attitude that evolves over time resulting from a relationship between store and consumer (Flavián et al., 2004). Approach behaviors refer to all positive actions that might be develop toward a setting, for example, intentions to explore or stay, while avoidance refers to the opposite (Mehrabian and Russell, 1974; Bitner, 1992; Gregg and Walczak, 2010). Real shopping outcomes are considered as behavioral contumer responses. This construction refers to quantitative measures such as time or money spent, products bought, etc.

Regarding mediator variables, involvement refers to the significant consciousness produced from product characteristics (Zaichkowsky, 1986). Atmospheric responsiveness is an environmental characteristic that influences users’ decisions on where and how to shop as well as the outcomes of the shopping experience (Eroglu et al., 2001, 2003). And, finally, perceived risk is the consumer’s level of uncertainty regarding the outcome of a purchase decision.

Respect to navigation structure, we propose compare two web manipulatios: “Free navigation structure” of the web site means that consumer can move easily through the web site (i.e., navigational bars and the same links to access the information in all sites, location link, and searching tool) and, consequently, the user is not controlled by web marketer with restrictive navigational bars. In contrast, in a web site with “hierarchical navigation structure,” the user only can use the “next” and “previous” navigational bars to move through the web site (i.e., user is exposed to restrictive navigational bars and, in consequence, its navigation is more difficult). Regarding music stimulus, we propose compare two web manipulations: Website “with music” versus website “non-music.” Finally, respect to “presentation of products” stimulus, we propose compare two web manipulations: “Moving images” (movement of photos in 360°, video, etc.) versus “static images.”

“One important feature of the new media that differs from traditional shopping channels is the absence of the experience about the online store visit and the unfeasibility of examining a product prior to purchase” (Lorenzo et al., 2007). Alba et al. (1997) indicate that a competitive advantage for marketers can be lead through an effective design of web site. But, how can marketers design web interfaces with high level of effectivity? Web atmospherics may offer insight into this question (Dailey, 2004).

Hoffman and Novak (1996) define network navigation as “the process of self-directed movement through the media involving non-linear search and retrieval methods that permit greater freedom of choice.” In traditional retail environments, consumers identify the spatial representations of the store’s design and recognize how products are grouped by their common characteristics or through orientation aids (i.e., displays, directory maps, store personnel, aisle markers, and so on) when they look for products (Titus and Everett, 1995).

Within the context of online behavior, we can introduce the usability concept. According to Nielsen (2000), the usability refers to the facility for the online navigation, and users are satisfied when it is easy to use by them. Therefore, usability is a quality attribute, and a key attribute to reach consumer satisfaction (Ranganathan and Ganapathy, 2002).

Dailey (2004) analyzes the impact of restrictive navigation cues as an explicit online webmosphere variable. She based on the theory of flow experience and the psychological reactance to explain that the restrictive navigation cues act as barriers that make users to be threated about the control over web navigation. This perceived barrier causes negative attitudes toward the web site and an avoidance behavior, which has negative consequences for the web marketer.

As other authors affirm, an easy navigational structure, such as a high task relevant (Eroglu et al., 2003), ease of use and usefulness (Childers et al., 2001), has a positive effect on the internal and behavioral consumer responses. Therefore, we propose the following hypothesis:

H_1-1_: An online shopping web with “free navigation structure” will affect in a more positive affective responses of users than “hierarchical navigation structure.”

The cognitive state in an online shopping refers issues related with how online consumers understand information provided on the screen to choose from different sites and products and the attitude toward the online store (Eroglu et al., 2003). Therefore, we considered the cognitive state as the consumers’ attitude and the knowledge attained during the shopping experience. So, we propose the following hypothesis:

H_1-2_: An online shopping web with “free navigation structure” will affect in a more favorable cognitive responses of users than “hierarchical navigation structure.”

Taking into account the double perspective of satisfaction meaning commented in Section “Sample and Procedure: Computer Experimental Design,” our research is focused on the attitudinal satisfaction perspective because it is more related to the purchase intention (Shankar et al., 2003). Specifically, satisfaction is understood as a global evaluation or attitude as a result of relationships between store and consumer (Flavián et al., 2004). For this reason, we will consider the satisfaction as an internal state which is affected by usability. So, the following hypothesis is proposed:

H_1-3_: An online shopping web with “free navigation structure” will influence on higher levels of satisfaction of users than “hierarchical navigation structure.”

Donovan et al. (1994) and Sherman et al. (1997) found that, in traditional contexts, the shoppers’ environmental perceptions affected their approach behaviors in the form of time and money spent, returning, store exploration, and so on. Lorenzo et al. (2007) observe that “within the online shopping environment, some works obtains similar approach/avoidance behavior depending on the perceived “store” environment and the mediating effects of individual traits and internal states (Eroglu et al., 2003).” In online environments, these effects have been less studied but literature indicates online atmospheric cues affect approach/avoidance responses. However, the relationship between both dimensions (i.e., stimuli and behavioral responses) is mediated by internal states for two cases: (a) For online environments with only utilitarian elements (Dailey, 2004); (b) and for online environments with utilitarian and hedonic elements (Childers et al., 2001; Eroglu et al., 2001, 2003). Having into consideration these above literature, we suggest the following hypothesis:

H_1-4_: An online shopping web with “free navigation structure” will affect in a more approach responses of users than “hierarchical navigation structure.”

Flavián et al. (2005) demonstrated a positive influence of web site usability on real shopping outcomes toward web site (mediated by confidence and satisfaction). In consequence, the following hypothesis is proposed:

*H_1-5_*: *An online shopping web with “free navigation structure” will influence on more positive real shopping outcomes than “hierarchical navigation structure.”*

On the other hand, music in the website is other non-verbal web technology stimulus. McMurray et al. (2008) argue that in traditional environments musical expectancy can influence on lower-level perceptual processes. Lorenzo et al. (2007) indicate that “creating a more enjoyable environment may require the use of more powerful web languages, and the inclusion of images, video, color, humor, sound, music, games, animation, and all of the other interactive aspects that could define an enjoyable experience. A technology oriented perspective that attempts to treat media shopping as cold information systems, rather than immersive, hedonic environments, is likely to be misguided, mainly for products with strong hedonic attributes, as can be the case of apparel (Childers et al., 2001).”

Eroglu et al. (2001, 2003) explain that sounds or music are low task-relevant cues, since they do not affect in a direct way the realization of the task. Nevertheless, music or sounds can help to create an atmosphere that has the possibility of making the shopping experience more pleasurable. In consequence, we propose the following hypothesis:

H_2-1_: An online shopping web with music will influence on more positive affective responses of users than a web environment without music.

Low task-relevant cues affect positively users’ cognitive states in online environments (Eroglu et al., 2001). In the literature there are several low-task relevant cues, but we will focus in music and its effect on users’ cognitive states (learning/knowledge and attitudinal process). Therefore, related with cognitive responses, the following hypothesis is proposed:

H_2-2_: An online shopping web with music will influence on more favorable cognitive responses of users than a web environment without music.

Customer’s satisfaction is defined as “a relative psychological state which is a result of purchase/consumption experience” (Vanhamme, 2000). Most of research demonstrate that store atmosphere influence on satisfaction and, in turn, behavioral responses (Bigné and Andreu, 2004) and, specifically, with hedonic webmosphere stimuli (Childers et al., 2001). So, we suggest the following hypothesis:

H_2-3_: An online shopping web with music will influence on more satisfaction of users than a web site without music.

In some studies where consumers were exposed to different tempo of background music within supermarkets, they obtained similar responses (Milliman, 1986; Oakes, 2003). Eroglu et al. (2001, 2003) demonstrated that consumers behave in different ways (approach/avoidance behaviors) depending on the online perceived store environment and the mediating effects of consumer internal states. They analyzed whether the online store information and the low-task relevant cues facilitate or impede the attainment of shopping goals and, in turn, whether the online shopper exhibited positive or negative behaviors toward the particular web site. Finally, they obtained that a rise of some atmospheric cue (high or low cue) increased the approach response of consumer. Because our intention is focused on study of specific atmospheric cue (i.e., music), we propose the following hypothesis:

H_2-4_: An online shopping web with music will affect in more approach responses of users than a web site without music.

Finally, regarding behavioral responses we proposed the following hypothesis, attending two groups of variables analyzed (loyalty and approach/avoidance behavioral). Specifically, as regards users’ loyalty toward online store after their visit in the web site is measured by Eroglu et al. (2003) as satisfaction measurement. However, according to above works, loyalty is considered as the consequence of the satisfaction (Zeithaml et al., 1996; Bigné and Andreu, 2004; Flavián et al., 2004) and loyalty means recommended the online store, better results, etc. In spite of this conceptual difference, the most works posit that atmospheric cues (specifically, low-task relevant cues according to Eroglu et al. (2001, 2003) affect positively real shopping outcomes toward store, although this relationship is mediated by consumers’ internal states. Taking everything into account, we suggest the following hypothesis:

H_2-5_: An online shopping web with music will affect in more positive real shopping outcomes than a web site without music.

Other non-verbal web technology stimulus, specifically hedonic dimensions, can be the presentation of product. According to Lorenzo et al. (2007), there are three kinds of animations depending on the technical characteristics that they use: animated gifs, flash format and video. In addition, Lorenzo et al. (2007) indicate that “the use of images and their animations as design elements within stores offers advantages and disadvantages depending on their use […]. Animating images has a potential effect on human’s periphery vision, specifically; it is very difficult to concentrate on reading a text if there is a revolving logo on the web page. In this case, it is more convenient to reduce the use of animations. However, if animation is used to make the purchase task easier, the use of animation will be more convenient.”

Schlosser (2003) examines how users process the images and texts offered by virtual media and state that “the image processing represents with a more relevant magnitude the object’s interactive effects on user intention –purchasing or browsing– than the processing of a verbal or in written speech. In fact, internal states as satisfaction variable is affected positively by animation elements on the website” (Childers et al., 2001; Eroglu et al., 2001, 2003; Adelaar et al., 2003; Wang et al., 2010).

The presentation of virtual products with animation design influence positively on consumer’s internal states and shopping outcomes (Dahan and Srinivasan, 2000; Eroglu et al., 2003; Koernig, 2003; Yoh et al., 2003; Hong et al., 2004; McKinney, 2004; Choura and Saber, 2005; Wang et al., 2010; Lian, 2011). In addition, Adelaar et al. (2003) demonstrated that verbal and visual presence –through the use of image and/or video– improve users’ emotional states. Therefore, we propose the following hypothesis:

H_3-1_: An online shopping web with “moving images” will affect in more positive affective responses of users than a web environment with “static images.”

In a similar vein, the introduction of “low task relevant cues” within the website, such as animation elements, influence favorable on consumer internal states (Dahan and Srinivasan, 2000; Childers et al., 2001; Eroglu et al., 2003; Hong et al., 2004). Moreover, Nielsen (2000) states that animations are efficacious when their transitions allow users to control them by perceptive system. So, the hypothesis proposed is:

H_3-2_: An online shopping web with “moving images” will influence on a more favorable cognitive responses of users than a web environment with “static images.”

The two-dimensional structure of a computer screen makes it difficult to understand a three-dimensional structure with only one image, regardless of its quality. So, animation is used to make the understanding of product’s spatial visualization which the users observe within the store easier. In this line, Hong et al. (2004) focus their research on the analysis of the use of Flash^®^ format on e-consumer behavior. As a result, the animation can increase the search for information if the animated element is relevant to the search task. Furthermore, Eroglu et al. (2003) state that the inclusion of this web attribute within the store facilitates the users’ information processing during the online purchase which, in turn, improves their satisfaction (McKinney, 2004). A lot of apparel websites lacks of movement of products and, even, static images, using in consequence only some descriptions about products. So, in order to analyze this type of web atmosphere cue, this research is focused in the comparison of static images versus a combination of eight pictures which offer an optical illusion of movement as well as the use of streaming.

The literature offers some works related to the relationship between web atmosphere and satisfaction. For instance, Childers et al. (2001) compared two types of web environments: one of them included only utilitarian elements (i.e., order and reception system) and the other one included also some hedonic characteristics (i.e., images of products with high resolution, background music, and interactive plays). Grobelny and Michalski (2015) also use an experimental study to analyze the influence of hedonic stimuli (color) on user men and women satisfaction. From Technology Acceptance Model, these authors obtained that both web dimensions have an equal role in the development of consumer’s attitudes and behavioral. In this sense, studies such as Eroglu et al. (2001, 2003) and Adelaar et al. (2003) also used different web experiments in their researches. From S-O-R Model, these authors obtained similar results (i.e., web elements such as images, and video, affect positively on internal and behavioral responses). In contrast, Kim and Stoel (2004) used the Loiaconno’s WebQualTM instrument to measure the effects of website quality dimensions (i.e., entertainment, web appearance, transaction capability, response time, informational fit-to-task, and trust) on shopper satisfaction. These authors obtained that only three dimensions (specifically, response time, transaction capability and informational fit-to-task) were important predictors of consumer satisfaction. To sum up, according to literature, animation elements of the web site affect positively on internal states, such as satisfaction (Childers et al., 2001; Eroglu et al., 2001, 2003; Adelaar et al., 2003; Hong et al., 2004). So, we proposed the following hypothesis:

H_3-3_: An online shopping web with “moving images” will influence on higher levels of satisfaction of users than a web environment with “static images.”

Schlosser (2003) examines how the users process the texts and images offered by online media, and demonstrate that “the image processing represents with a more relevant magnitude the object’s interactive effects on user intention –purchasing or browsing– than the processing of a verbal or in written speech. So, the product’s interactivity causes intense mental images which will influence more strongly on user’s intention than in their cognitive processing” (Lorenzo et al., 2007). In the same line, Childers et al. (2001) state that the functional aspects such as usability, navigational structure, etc., cause strong influences on the e-consumer responses. Moreover, the rest of web elements not functional such as music, color, images with high resolution, etc., are equally relevant elements which contribute to the shaping of consumer attitudes and behavior (i.e., opinions about the store and unexpected purchase behavior). Based on previous literature, in webs which includes animations, costumer’s show more positive internal states, which improve their shopping experience and, in consequence, their approach responses. Having into consideration the above literature, we propose the following hypothesis (Lorenzo et al., 2007):

H_3-4_: An online shopping web with “moving images” will affect in more approach responses of users than a web environment with “static images.”

Eroglu et al. (2003) argue that the inclusion of entertainment within the store raises the consumer’s positive behavior toward the website. On the other hand, Koernig (2003) affirms that the higher the product’s tangibility level represented through the screen, the more positive behavioral response. Khakimdjanova and Park (2005) affirm that the use of online visual display features that allow viewing the garment from different angles can help potential online consumers to make a purchase decision.

In conclusion, according to literature, the use of animation designs for presenting products influence positively on consumer’s internal states and shopping real outcomes (Dahan and Srinivasan, 2000; Adelaar et al., 2003; Eroglu et al., 2003; Koernig, 2003; Yoh et al., 2003; Hong et al., 2004; McKinney, 2004; Choura and Saber, 2005). In addition, Argyriou (2012) demonstrated that website characteristics do not affect revisit intentions directly but through the vividness of mental images that consumers hold of the website as a whole. Vivid mental website imagery is stimulated by animation and facilitated by individual tendencies to put faith in intuitive rather than rational thinking. Then, it is proposed that (Lorenzo et al., 2007):

H_3-5_: An online shopping web with “moving images” will affect in more positive real shopping outcomes than a web environment with “static images.”

Regarding mediator variables, Baron and Kenny (1986) analyze the differences between the properties’ mediator and moderator variables. In general, mediator variables explain how external physical events take on internal psychological significance. Whereas moderator variables specify when certain effects will hold, mediators speak about how or why such effects occur (Lorenzo et al., 2007). Taking into account this distinction, in this research were studied three variables (i.e., involvement, atmospheric responsiveness, and perceived risk) as possible mediator between constructions analyzed.

Based on Eroglu et al.’s (2003) work, the relationship between virtual atmosphere and internal states is mediated by user personal characteristics (e.g., involvement and atmospheric responsiveness).

Zaichkowsky (1986) defines product involvement as the relevant consciousness caused by product characteristics. Moreover, according to Goldsmith and Emmert (1991), the higher the level of product involvement, the higher the search for product information. Therefore, product involvement is an important variable in consumer behavior.

Then, variables such as involvement with the product (Keng et al., 2012), and specifically with virtual purchase (Hernández et al., 2010), experience with the new media and advertisement (Fortin and Dholakia, 1999), should affect electronic shopping (Yoh et al., 2003). In fact, atmospheric responsiveness is an environmental characteristic that influences consumers’ decisions -where and how to shop- as well as the shopping experience (Eroglu et al., 2001, 2003). Lorenzo et al. (2007), based on Huang’s (2006) research explain that “flow and involvement are known as motivational constructs (Csikszentmihalyi, 1975). The concept behind involvement lies in personal relevance, regardless of whether the locus of personal relevance resides in the consumer or the situation (Celsi and Olson, 1988). Enduring and situational involvement differ in aspects such as the temporal pattern of occurrences, the motivations and the benefits sought (Huang, 2006). Enduring involvement is intrinsically motivated, whereas situational involvement is extrinsically motivated (Kapferer and Laurent, 1985). So, consumers who are enduringly involved are looking for hedonic benefits, whereas consumers who are situational involved are engaged in goal-directed behaviors (Hoffman and Novak, 1996).”

Otherwise, perceived risk also influence on consumer behavior depending on web stimulus (Yoh et al., 2003; Smith and Sivakumar, 2004; Park and Stoel, 2005), so it is considered as a mediator variable. Koernig (2003) affirm that if the tangibility of the service is increased in the online environment, the perceived risk related to the activity will decrease. In addition, Slovic et al. (1980) argue that the presence of images with low quality within virtual stores cause a negative influence on consumer behavior, and also it will increase the perceived risk. On the other hand, Chang and Wu (2012) study the effect of perceived risk by analyzing the moderating effects of decision-making style, it means involvement or heuristics, in the virtual context, obtaining that perceived risk toward a website influences on purchasing intention through cognition, and this affects on attitudes. Within this line, Keng et al. (2012) analyzed the moderating roles of needing for touching and implication with the product.

Based on previous literature, four additional hypotheses for each three webmosphere analyzed stimuli are proposed respect to mediator variables –two of them related to involvement– as follow (a: Navigational structure; b: Music; c: Presentation of products):

H4a,b,c: Involvement with apparel will have a positive mediator effect between the three different web stimuli (i.e., music, navigational structure and presentation of products) and online user responses (i.e., internal and behavioral responses)
H5a,b,c: Involvement with virtual shopping will have a positive mediator effect between the three different web stimuli and online user responses
H6a,b,c: Atmospheric responsiveness will have a positive mediator effect between the three different web stimuli and online user responses
H7a,b,c: Perceived risk will have a positive mediator effect between the three different web stimuli and online user responses

**FIGURE 1:**
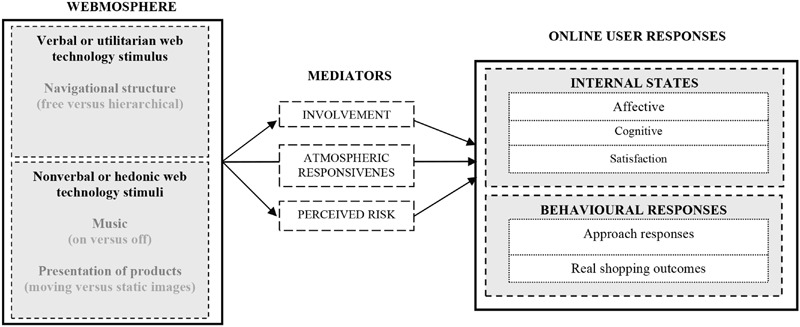
**Stimulus-Organism-Response (S-O-R) proposed model to measure the effects of webmosphere non-verbal and verbal communication stimuli on online user responses**.

To test above hypotheses, an extended model is proposed (**Figure 1**) based on the S-O-R paradigm. The direct effects between specific web technological attributes related to non-verbal (i.e., hedonic: music and visualization of products) and verbal (i.e., utilitarian: navigational structure) web dimensions on online user responses (i.e., internal states and behavioral responses) were analyzed, as well as examining the influence of mediator variables (i.e., involvement, atmospheric responsiveness, and perceived risk) between both constructs (i.e., web stimuli and online user responses).

## Materials and Methods

### Sample and Procedure: Computer Experimental Design

A 2 (“free” versus “hierarchical” navigational structure) × 2 (“on” versus “off” music) × 2 (“moving” versus “static” images) between-subjects computer experimental design was used to carry out the research. Similar types of computer experimental designs with more/less, different/similar web stimuli have been analyzed in previous studies (e.g., Eroglu et al., 2001, 2003; Dailey, 2004; Choura and Saber, 2005; Nesset et al., 2011; Chang and Wu, 2012). A fictitious store was created for this research (named E-Fashion) to avoid the effects of prior experience with a e-retailer. That virtual shop offered fashion apparel (clothes and accessories) for men and women, in two designs (casual and formal). To standardized products to compare men and women, the number of clothes and accessories were the same for the two genders. To create the content of the virtual shop, we based on a homepage (Schneiderman, 1998; Nielsen, 2000), which incorporates the similar links and web sites as other virtual apparel shops.

In sum, the experimental design consisted on the double combination between three types of web stimuli:

*(1) Navigation structure stimulus*. We used two different web navigational structure used in this study:

• “Free navigation structure” of the website means that costumer can move easily through the website. For that, the following tools have been included within this experimental condition: “home link,” “location pattern,” “searching window,” “lateral menus in all webpages of the site”). Consequently, the user is not controlled by marketer with restrictive navigational bars.
• “Hierarchical navigational structure” means that user only can use the “previous” and “next” navigational bars to move through the website. In consequence, the previous tools (“home link,” “location pattern,” “searching window,” “lateral menus in all webpages of the site”) have been eliminated of this experimental condition in order to create more restrictive navigation to user.

*(2) Music stimulus*. Two different music web stimuli were used in this study:

• “On-music” within the webpage. In this case, the music was included within the experiment. Music was tested previously to analyze which kind of music was preferred by consumers, using music that already existed. The kind of music included was instrumental and very dynamic, but it was not readily identifiable with any particular singer or specific group. It was used music of the album Traveler’s guide by Jahzzar, which is licensed under a Attribution-ShareAlike 3.0 International License.
• “Off-music.” In this case, the music was eliminated of the experiment.

*(3) Presentation of product stimulus*. We used two different presentations of products (Martin et al., 2005; Lorenzo et al., 2007):

• “Static images” web design presents the possibility of seeing frontal images of all products and a window with static photos of different models showing several outfits.
• “Moving images” web design allows consumers 360° visualization of products using gif images according to animation definition defined by Dahan and Srinivasan (2000) and Sundar and Kalyanaraman (2004), as well a window with videos in which the models show the fashion apparel of the store (Choura and Saber, 2005; Nesset et al., 2011).

As a result of combination the three webmospheric manipulations (2 × 2 × 2), eight different websites were defined:

(1) Hierarchical navigation + on music + moving images;
(2) Hierarchical navigation + off music + moving images;
(3) Hierarchical navigagion + on music + static images;
(4) Hierarchical navigation + off music + static images;
(5) Free navigation + on music + moving images;
(6) Free navigation + off music + moving images;
(7) Free navigation + on music + static images;
(8) Free navigation + off music + static images.

In all experimental designs, the content of webpages is the same (i.e., company description, products description, shopping car, security and privacy policy, promotions, fictitious money to buy, link to register, new arrivals and sale section, contact and search engine), excepting the changes of stimuli indicated in each manipulation. From both links previously indicated, according to the each experiment, gifs animated or static images (in the case of “presentation of product” experimental design), and music or not (in the case of “music” experimental design) were included.

Before starting the experiment, a pre-test was conducted to a different group to ensure that subjects’ responses give us different perception by inclusion of the above atmospheric manipulations.

The final sample consisted of 400 participants who were divided into eight groups (two of them with the navigational structure manipulation, two of them with music manipulation, two of them with presentation manipulation). Participants were randomly assigned to one of these experimental conditions. All members of each group were undergraduate students (18–25 years old, and half of the same were men and the other half were women), due to it was a group with enough experience and positive predisposition to the Internet as to carry out this experiment in adequate way (Ozok and Wei, 2010). All groups were exposed same external environment conditions (place and space) throughout the same day. Besides these aspects, all experiments were checked under same conditions explained above: time (50 min, since in the pre-test was demonstrated that people spent on average this time until take a final decision), money (each subject had 200€; of budget, which is an average of the total budget that they usually have, according with a previous exploratory analysis) and elements of web site. In order to analyze an equilibrated model, each group was consisted of 50 subjects.

After participants finished the experimental task they were complete a final online questionnaire which includes affective, cognitive and satisfaction measures (i.e., user internal states), behavioral responses (i.e., approach responses toward the website), and mediators variables (i.e., involvement with apparel and online purchase, atmospheric responsiveness, and perceived risk). All items, kinds of scales and sources used to generate the online questionnaire are showed in **Table 1**. Participants of eight experiments were asked by the same questionnaire, it means, eight groups of questions corresponding to each scale, which it was a total of 36 items.

**Table 1:** Measurement of variables.

Variables	Items	Kind of Scale (five points)	Source of the Scale
**Internal states**			
Affective	*Attitude towards online purchase:* ⋅ Disappointed/appointed ⋅ Unfavorable/favorable ⋅ Negative/positive *Emotion toward online purchase:* ⋅ Bored/non-bored ⋅ Unhappy/Happy ⋅ Unaroused/aroused	Semantic differential question	Mehrabian and Russell, 1974; Eroglu et al., 2003; Ivonin et al., 2013
Cognitive	*Believes about online apparel purchase:* ⋅ Easy ⋅ Cheap ⋅ Enjoy *Learning and knowledge obtained:* ⋅ Learning about new media for shopping ⋅ Knowledge about online purchase	Likert scale	Lorenzo et al., 2007; Schlosser, 2003
Satisfaction	*General satisfaction with the visit:* ⋅ Browsing satisfaction ⋅ Security satisfaction ⋅ Satisfaction with the shopping experience *Satisfaction with design:* ⋅ Design of the store is liked by users ⋅ Satisfaction with the presentation of products within the online store ⋅ Navigational elements help users move across the website	Likert scale	Anderson and Srinivasan, 2003; Eroglu et al., 2003; Cristóbal, 2005; Flavián et al., 2004, 2005; [Bibr B73][Bibr B60] SUMI scale
**Behavioral Responses**			
Approach responses	*Behavior intention and opinion about the website:* ⋅ Revisiting the store ⋅ Recommendation of the store to other people ⋅ User would have spent more money in the website ⋅ User would have spent more time in the website ⋅ Navigational elements attract attention to users *No expected shopping behavior:* ⋅ Users buy more products than planned previously ⋅ Users spend more money than planned previously	Probability scale	Zeithaml et al., 1996; Sherman et al., 1997; Eroglu et al., 2003; Bigné and Andreu, 2004; Dailey, 2004; Flavián et al., 2004, 2005; Gallarza and Gil, 2006; Lorenzo et al., 2007; Ozok and Wei, 2010; Chen and Teng, 2013
Real shopping outcomes	⋅ Time spent during the visit ⋅ Products bought ⋅ Money spent	Click-through	Eroglu et al., 2003
**Mediators variables**			
Involvement	*Involvement with apparel:* ⋅ I like going shopping to see or buy apparel ⋅ I like wearing fashion apparel *Involvement with online purchase:* ⋅ I like buying on the Internet ⋅ It is enjoyable buying on the Internet	Likert scale	Kapferer and Laurent, 1985; Yoh et al., 2003; Keng et al., 2012; Kim, 2012
Atmospheric responsiveness	⋅ When visit online apparel stores I usually perceive the design of website ⋅ The design of online stores influence on my possible visit to them	Likert scale	Eroglu et al., 2003
Perceived risk	⋅ Internet transmits security in the purchases ⋅ It is safe to buy apparel without taking them previously ⋅ I would buy other products such as tickets, books, digital music, computer goods…	Likert scale	Anderson and Srinivasan, 2003; O’Cass and Fenech, 2003; Flavián et al., 2004, 2005; Gregg and Walczak, 2010; Kim, 2012

Finally, based on Lorenzo et al. (2007), the web-based tool created for this research involved an automatic tracking process. This software was able to track and record all click-troughs and times related with the browsing behavior during the experiment to obtain behavioral responses in the proposed model (i.e., real shopping outcomes) to clarify the data captured by the software.

It is worth noting that that this study was carried out in accordance with the current ethical and legal recommendations about privacy of personal data. Throughout the whole research we take into account also the ICC/ESOMAR International Code on Market and Social Research practices and norms.

### A Model of Web Technology Effects on User Responses. Measurement of Variables

We propose an extended model (**Figure 1**) based on the S-O-R paradigm (Mehrabian and Russell, 1974), to test the influence of three specific atmospheric cues on online shopper responses. The main objective is to analyze the differences of response between the experimental groups, as well as examining the influence of mediator variables between the constructs. The analyzed variables in each construct are showed in **Table 1**.

### Statistical Techniques

Regarding the statistical techniques used in this research, as our major objective is to analyze whether or not there were significant differences of behavior between the groups and the effects of each online atmospheric manipulation cues on five types of dependents constructs respect to the consumer: Affective, cognition and satisfaction (as internal states), and approach/avoidance responses and real shopping outcomes (as consumer behavioral responses), a multivariate analysis of variance (MANOVA) is used to test this part. Additionally, a multivariate analysis of covariance (MANCOVA) is used to analyze the mediator effect of involvement, atmospheric responsiveness, and perceived risk factors between web verbal and non-verbal communication stimuli and user responses.

Previously, as the variable used as dependents and mediators are latent variables, it means, non-observable variables measured through scales (see **Table 1**), we used exploratory factor analysis to describe variability among observed, correlated variables in terms of a potentially lower number of unobserved variables called factors. Factor analysis searches for such joint variations in response to unobserved latent variables.

## Results

In **Tables 2** and **3** are showed the data obtained after factorial analysis in each construct used, which are necessary to be used in the multivariate analysis (MANOVA and MANCOVA). The first step of the analysis is to test the model fit, that is, if it is a good idea to proceed with a factorial analysis for the data, which is showed in **Table 2**. The next step is to analyze the factors obtained from the factorial analysis, which are showed in **Table 3**.

**Table 2:** Association level indicators between analyzed variables within the model.

	Online user internal states			
	Affective		Cognitive	Satisfaction
Correlation matrix	Correlated variables		Correlated variables	Correlated variables
Correlation matrix determinant	0.016		0.193	0.059
Kaiser-Meyer-Olkin index	0.894		0.687	0.872
Barlett’s sphericity proof	1633.101 *p* < 0.001		652.148 *p* < 0.001	1123.731 *p* < 0.001
Anti-image correlation matrix	Partial correlation		Reduced partial correlation	Partial correlation
Measure of sample adequacy	Coefficient between 0.85 and 0.91		Coefficient between 0.50 and 0.75	Coefficient between 0.85 and 0.89

**Behavioral responses**				
**Approach responses**				

Correlation matrix		Correlated variables		
Correlation matrix determinant		0.075		
Kaiser-Meyer-Olkin index		0.748		
Barlett’s sphericity proof		1027.201 *p* < 0.001		
Anti-image correlation matrix		Reduced partial correlation		
Measure of sample adequacy		Coefficients between 0.58 and 0.88		

**Mediator variables**				
**Involvement; atmospheric responsiveness; perceived risk**				

Correlation matrix		Partial correlated variables		
Correlation matrix determinant		0.354		
Kaiser-Meyer-Olkin index		0.549		
Barlett’s sphericity proof		410.489 *p* < 0.001		
Anti-image correlation matrix		Partial correlation		
Measure of sample adequacy		Coefficients between 0.49 and 0.58		

**Table 3:** Factorial analyses for each construct (variables and covariates).

Affective variables (a)	Factors			
	Attitude		Emotion	
Dissapointed/appointed	**0.822**			
Unfavorable/favorable	**0.883**			
Negative/positive	**0.860**			
Bored/non-bored			**0.921**	
Unhappy/Happy			**0.644**	
Unaroused/aroused			**0.607**	
Eigenvalues of the factors	4.215		0.624	
% Explained variance	70.250		10.403	
Cronbach alpha	0.9061		0.8308	

**Cognitive variables (b)**	**Factors**			
	**Believes about online apparel purchase**		**Learning and knowledge obtained**	

Easy	**0.873**			
Cheap	**0.909**			
Enjoy	**0.866**			
Learning about new media for shopping			**0.847**	
Knowledge about online purchase			**0.838**	
Eigenvalues of the factors	2.370		1.415	
% Explained variance	47.398		28.296	
Cronbach alpha	0.8595		0.5972	

**Satisfaction variables (c)**	**Factors**			
	**General satisfaction with the visit**		**Satisfaction with the design**	

Browsing satisfaction	**0.835**			
Security satisfaction	**0.709**			
Satisfaction with the shopping experience	**0.863**			
Design of the store is liked by users			**0.726**	
Satisfaction with the presentation of products within the online store			**0.597**	
Navigational elements help users move across the website			**0.871**	
Eigenvalues of the factors	3.713		0.667	
% Explained variance	61.887		11.123	
Cronbach alpha	0.8419		0.8595	

**Behavioral responses**	**Factors**			
**Approach responses to the website (d)**	**Behavior intention and opinion about the website**		**No expected shopping behavior**	

Revisiting the store	**0.850**			
Recommendation of the store to other people	**0.841**			
User would have spent more money in the website	**0.731**			
User would have spent more time in the website	**0.796**			
Navigational elements attract attention to users	**0.464**			
Users buy more products than planned previously			**0.892**	
Users spend more money than planned previously			**0.911**	
Eigenvalues of the factors	3.163		1.466	
% Explained variance	45.181		20.941	
Cronbach alpha	0.8112		0.8114	

**Mediator variables or covariates (e)**	**Factors**			
	**Involvement with the online shopping**	**Involvement with the apparel**	**Atmospheric responsiveness**	**Perceived risk with the virtual shopping**

I like buying on the Internet	**0.846**			
It is enjoyable buying on the Internet	**0.796**			
I like going shopping to see or buy apparel		**0.848**		
I like wearing fashion apparel		**0.854**		
When visit online apparel stores I usually perceive the design of website			**0.760**	
The design of online stores influence on my possible visit to them			**0.857**	
Internet transmits security in the purchases				**0.603**
It is safe to buy apparel without taking them previously				**0.710**
I would buy other products such as tickets, books, digital music, computer goods…				**0.577**
Eigenvalues of the factors	2.052	1.442	1.199	1.021
% Explained variance	22.798	16.021	13.317	11.346
Cronbach alpha	0.6400	0.6388	0.5290	0.1942

*(a), (b), (c), (d), (e) Extraction Method: Principal Components Analysis; Rotation Method: Kaiser Varimax Normalization; (a), (b), (c) The rotation has converged in 3 iterations; (d) The rotation has converged in 4 iterations (e) The rotation has converged in 5 iterations.*

*Bold values reflect which items load on each factor.*

Specifically, in **Table 2** is showed the association level indicators between analyzed variables which compose the different constructs of our model. For example, “affective” construct shows that its variables are correlated, there is no multicollinearity since the correlation matrix determinant is greater than 0.00001, the K-M-O index is greater than 0.8 and Bartlett’s test of sphericity is highly significant. These indicators, together with the measure of sample adequacy (>0.5), indicate good association level of items on its construct (Iacobucci, 1994; Hair et al., 2006). In the rest of construct (i.e., “cognitive,” “satisfaction,” “behavioral responses,” and “mediator variables”) the results are similar, meaning these results a good association level of items on their constructs. Lower levels are obtained in the “mediator variables” due to the inclusion of different types of covariates with different meanings (i.e., involvement, atmospheric responsiveness, perceived risk). Nevertheless, the global statistics indicators are acceptable (i.e., K-M-O index 0.549; *p*-value < 0.01; measure of sample adequacy with coefficients between 0.49 and 0.58). It means a good association level between mediator variables.

In **Table 3** is showed the factorial analyses for each analyzed construct (i.e., “affective,” “cognitive,” “satisfaction” as internal variables construct; “approach responses” as behavioral responses construct; and “mediator variables or covariates” construct). Each obtained factor shows the name of items with which is composed. For example, “affective variables construct” is composed by two factors: “Attitude” and “Emotion.” In this case, each factor is composed by three items. The rest of constructs are composed by two factors, excepting “mediator variables” construct which is composed by four factors.

In the case of “real shopping outcomes” (within “behavioral responses” construct) the factorial analysis is not possible due to the variables are uncorrelated. These variables were obtained through click-through -it has been indicated in **Table 1**- carried out by users during their visit within online retail store. Nevertheless, the variables are used in the ANOVA and ANCOVA post analysis (Davino and Romano, 2014).

For each factor has been analyzed the Eigenvalues, the percentage of explained variance, and specially, the Cronbach Alpha index which measures the level of reliability of factor. In all cases, the eigenvalue is greater than 1.000, the level of explained variance is higher 0.5 and the Cronbach alpha near 1.000 (Cronbach, 1970). It means a good fit of the factorial analysis (Iacobucci, 1994; Hair et al., 1999).

In sum, as we can observe in **Tables 2** and **3**, the Cronbach Alpha is used as reliability measurement of all analyzed constructs (Cronbach, 1970). Moreover, the Wilks’ Lambda statistic was used to test the global significance (Iacobucci, 1994; Hair et al., 1999), using the F-Snedecor statistic and, as accepted confidence statistic level, 95% (*p*-value = 0.05) and 90% (*p*-value = 0.1), as it is showed in **Tables 4** and **5**. In consequence, validity and reliability obtained from this factorial analysis show us the suitability of its application for the next multivariate analysis. Moreover, the content validity is obtained through the application of literature in the development of our proposed model.

**Table 4:** Global contrast: MANOVA vs. MANCOVA on online users’ internal states.

Factors		Effects	Λ Wilks		F Snedecor		Significance		Hypotheses		
			MAN	MANC	MAN	MANC	MAN	MANC	MAN	MANC	
Internal states	Affective	NAV	0.984	0.982	3.124	3.461	0.045^∗^	0.032^∗^	H_1-1_		
		MUS	1.000	1.000	0.071	0.068	0.932	0.935	H_2-1_		
		Present	0.997	0.997	0.581	0.611	0.560	0.543	H_3-1_		
		INV-A		0.916		17.739		<0.001^∗^		H_4_	a
		INV-OS		0.961		7.856		<0.001^∗^		H_5_	
		AR		1.000		0.044		0.957		H_6_	
		PR		0.988		2.307		0.101 ^∗∗^		H_7_	
	Cognitive	NAV	0.966	0.964	6.872	7.263	<0.01^∗^	<0.01^∗^	H_1-2_		
		MUS	0.991	0.988	1.816	2.374	0.164	0.094^∗∗^	H_2-2_		
		Present	0.992	0.995	1.557	0.903	0.212	0.406	H_3-2_		
		INV-A		0.917		17.613		<0.001^∗^		H_4_	b
		INV-OS		0.946		11.043		<0.001^∗^		H_5_	
		AR		0.656		101.529		<0.001^∗^		H_6_	
		PR		0.979		4.096		0.017^∗^		H_7_	
	Satisfaction	NAV	0.948	0.948	10.677	10.673	<0.001^∗^	<0.001^∗^	H_1-3_		
		MUS	0.993	0.991	1.446	1.748	0.237	0.175	H_2-3_		
		Present	0.986	0.986	2.707	2.669	0.068^∗∗^	0.071^∗∗^	H_3-3_		
		INV-A		0.925		15.767		< 0.001^∗^		H_4_	c
		INV-OS		0.968		6.342		< 0.001^∗^		H_5_	
		AR		0.998		0.292		0.747		H_6_	
		PR		0.991		1.708		0.183		H_7_	

***^∗^***p*-value < 0.05 confidence level: 95%. NAV, navigational structure; MUS, music; PRESENT, presentation of products; INV-A, involvement with apparel; INV-OS, involvement with online shopping; AR, atmospheric responsiveness; PR, perceived risk; MAN, MANOVA; MANC, MANCOVA. ^∗∗^*p* < 0.1.*

**Table 5:** Global contrast: MANOVA vs. MANCOVA on online users’ behavioral responses.

Factor and variables		Effects	Λ Wilks		F Snedecor		Significance		Hypotheses		
			MAN	MANC	MAN	MANC	MAN	MANC	MAN	MANC	
Behavioral responses	Approach responses (factor)	NAV	0.999	0.999	0.195	0.166	0.823	0.847	H_1-4_		
		MUS	0.994	0.994	1.121	1.220	0.327	0.296	H_2-4_		
		Present	0.982	0.983	3.491	3.282	0.031^∗^	0.039^∗^	H_3-4_		
		INV-A		0.951		10.074		<0.001^∗^		H_4_	d
		INV-OS		0.985		2.979		0.052^∗^		H_5_	
		AR		0.989		2.136		0.120		H_6_	
		PR		0.977		4.654		0.010^∗∗^		H_7_	
	Real shopping outcomes (variables)	NAV	0.997	0.996	0.407	0.534	0.748	0.659	H_1-5_		
		MUS	0.994	0.993	0.801	0.946	0.494	0.418	H_2-5_		
		Present	0.912	0.911	12.555	12.532	<0.001^∗^	<0.001^∗^	H_3-5_		
		INV-A		0.975		3.365		0.019^∗^		H_4_	e
		INV-OS		0.989		1.400		0.242		H_5_	
		AR		0.999		0.153		0.928		H_6_	
		PR		0.990		1.295		0.276		H_7_	

***^∗^***p*-value < 0.05, **^∗∗^***p*-value < 0.1.*

A comparative analysis (MANOVA vs. MANCOVA) is developed in order to compare the effects of web stimuli on user responses without (MANOVA) and with (MANCOVA) the inclusion of mediator variables, respectively. Each significance item (*p*-value < 0.05 and 0.1) confirm the webmospheric cues preferred by users. The global contrasts are showed in **Table 5** (internal states) and **Table 6** (behavioral responses).

**Table 6:** Individual contrasts: ANOVAS vs. ANCOVAS on consumer responses with the NAVIGATIONAL STRUCTURE as VERBAL communication stimuli.

Verbal webmospheric cue: navigational structure (“hierarchical vs. free”)								
**Consumer responses**		**Factors and ítems**	***F***		**Significance**		**Mean differences (“hierarchical – free”)**	
			**ANOVA**	**ANCOVA**	**ANOVA**	**ANCOVA**	**ANOVA**	**ANCOVA**

Internal states	Affective (factors)	*Attitude*	0.462	0.869	0.497	0.352	-0.068	-0.091
		*Emotion*	5.812	5.804	0.016	0.016	-0.240^∗^	-0.235^∗^
	Cognifive (factors)	*Believes about online apparel purchase*	3.940	3.666	0.048	0.056	-0.197^∗^	-0.154^∗^
		*Learning and knowledge obtained*	9.572	10.374	0.002	0.001	-0.307^∗^	-0.303^∗^
	Satisfaction (factors)	*General satisfaction with the visit*	2.225	1.964	0.137	0.162	0.149	0.136
		*Satisfaction with the design*	18.999	19.832	< 0.001	< 0.001	-0.424^∗^	–0.430^∗^
Behavioral responses	Approach responses (factors)	*Behavior intention and opinion about the website*	0.083	0.025	0.773	0.874	0.029	0.016
		*No expected shopping behavior*	0.308	0.311	0.579	0.578	-0.056	–0.055
	Real shopping outcomes (variables)	*Time spent during the visit*	0.139	0.192	0.709	0.662	0.400	0.469
		*Products bought*	0.782	1.120	0.377	0.291	-0.230	-0.277
		*Money spent*	0.383	0.616	0.537	0.433	-3.980	-5.076

***^∗^***p*-value < 0.05. ANOVA, variance univariable analysis; ANCOVA, covariance univariable analysis.*

In order to clarify the specific impact of each stimulus on each consumer response factor, the individual contrasts are showed for each web manipulation in **Table 6** (i.e., navigational structure), **Table 7** (i.e., music) and **Table 8** (i.e., presentation of products), through ANOVA (univariate analysis of variance) and ANCOVAS (univariate analysis of covariance) analysis (Davino and Romano, 2014).

**Table 7:** Individual contrasts: ANOVAS vs. ANCOVAS on consumer responses with the MUSIC as NON-VERBAL communication stimuli.

Non-verbal webmospheric cue: music in the retail online store (“off vs. on music”)								
**Consumer Responses**		**Factors and items**	***F***		**Significance**		**Mean differences (“Off - On music”)**	
			**ANOVA**	**ANCOVA**	**ANOVA**	**ANCOVA**	**ANOVA**	**ANCOVA**

Internal states	Affective (factors)	*Attitude*	0.132	0.127	0.716	0.722	-0.036	-0.035
		*Emotion*	0.010	0.005	0.922	0.942	0.010	-0.007
	Cognifive (factors)	*Believes about online apparel purchase*	0.593	0.218	0.442	0.641	-0.077	-0.038
		*Learning and knowledge obtained*	2.991	4.455	0.085	0.035	-0.171^∗∗^	-0.198^∗^
	Satisfaction (factors)	*General satisfaction with the visit*	0.110	0.159	0.740	0.691	–0.033	–0.039
		*Satisfaction with the design*	2.804	3.294	0.095	0.070	-0.163^∗∗^	-0.175^∗∗^
Behavioral responses	Approach responses (factors)	*Behavior intention and opinion about the website*	1.131	1.498	0.288	0.222	-0.106	-0.120
		*No expected shopping behavior*	1.120	0.978	0.291	0.323	0.106	0.098
	Real shopping outcomes (variables)	*Time spent during the visit*	0.570	0.790	0.451	0.375	-0.810	-0.948
		*Products bought*	0.591	0.681	0.442	0.410	0.200	0.215
		*Money spent*	1.325	1.506	0.250	0.221	7.406	7.918

***^∗^***p*-value < 0.05, **^∗∗^***p*-value < 0.1.*

**Table 8:** Individual contrasts: ANOVAS vs. ANCOVAS on consumer responses with the PRESENTATION OF PRODUCTS as NON-VERBAL communication stimuli.

Non-verbal webmospheric cue: presentation of products (“static vs. moving images”)								
**Factors and ítems**		**Factors and ítems**	***F***		**Significance**		**Mean differences(“Static - Moving images”)**	
			**ANOVA**	**ANCOVA**	**ANOVA**	**ANCOVA**	**ANCOVA**	**ANCOVA**

Internal states	Affective (factors)	*Attitude*	0.132	0.090	0.716	0.765	-0.036	-0.029
		*Emotion*	1.036	1.098	0.309	0.295	-0.101	-0.102
	Cognifive (factors)	*Believes about online apparel purchase*	3.016	1.651	0.083	0.200	0.173^∗∗^	0.103
		*Learning and knowledge obtained*	0.083	0.120	0.774	0.729	0.029	0.032
	Satisfaction (factors)	*General satisfaction with the visit*	0.672	0.654	0.413	0.419	-0.082	-0.078
		*Satisfaction with the design*	4.805	4.576	0.029	0.033	-0.213^∗^	-0.206^∗^
Behavioral responses	Approach responses (factors)	*Behavior intention and opinion about the website*	6.672	6.224	0.010	0.013	-0.258^∗^	-0.244^∗^
		*No expected shopping behavior*	0.324	0.323	0.569	0.570	-0.057	-0.056
	Real shopping outcomes (variables)	*Time spent during the visit*	18.963	19.272	<0.001	<0.001	-4.670^∗^	-4.677^∗^
		*Products bought*	20.232	19.535	<0.001	<0.001	-1.170^∗^	-1.151^∗^
		*Money spent*	18.979	18.404	<0.001	<0.001	-28.028^∗^	-27.643^∗^

***^∗^***p*-value < 0.05, **^∗∗^***p*-value < 0.1.*

### Effects of Verbal Communication Stimulus (Utilitarian Dimension) on User Responses: Navigational Structure

The global contrast MANOVA test (**Table 4**) shows significant differences between groups analyzed regarding the relationship between this verbal web stimulus and all internal states factors (*p*-value < 0.05). In the ANOVA test (**Table 6**) we can see the positive effect of “free navigation” on user’s internal states. Specifically, the factors like emotion (as affective internal state), believes and learning/knowledge (as cognitive states), and satisfaction with the design (as satisfaction state) are related positively with the “free navigation” as verbal communication stimulus.

In **Table 4**, MANCOVA test includes four mediator factors in which we can see the significance impact of involvement (with apparel and with online shopping) on all of three internal states. Based on **Table 6**, the ANCOVA test shows that users who were exposed to an online shopping environment with the “free navigation” show more favorable internal responses than those who were exposed to the “hierarchical navigation” during their visit within online retail store (the factors attitude and general satisfaction with the visit are not significantly different between both navigational structure including involvement as mediator factors).

Regarding atmospheric responsiveness as mediator factor, **Table 4** shows the inexistence of significant differences respect to affective and satisfaction internal states (*p*-value = 0.957 and 0.747, respectively). Nevertheless, atmospheric responsiveness influence on cognitive states, and we can affirm that users prefer free navigation.

Finally, respect to perceived risk mediator factor, **Table 4** shows significant influence on affective and cognitive internal states, whose preference is focus on “free navigation,” but perceive risk do not influence on satisfaction.

Regarding the effects on behavioral responses, **Table 5** shows the inexistence of significant differences between analyzed groups exposed to “hierarchical” versus “free” navigational structure on behavioral responses. The impact of internal variables as mediator factors between this non-verbal stimuli and behavioral responses has not been analyzed. It could be the cause of this non-significant effect. The univariate test of **Table 6** does not show significant difference in any analyzed factors related to behavioral responses. Moreover, the inclusion of mediator factors (involvement, atmospheric responsiveness, perceived risk) do not influence on the behavioral responses.

### Effects of Non-verbal Communication Stimulus (Hedonic Dimension) on User Responses: Music in the Website

**Table 4** shows significant differences between this non-verbal stimulus and internal states, specifically respect to cognition states. Moreover, analyzing the mean differences between both types of manipulations (“on music” versus “off music”), in the **Table 7** we can observe that users who were exposed to an online shopping environment with the “on music” web designs show more favorable learning and knowledge of the website and higher satisfaction with the design than those who were exposed to “off music” conditions within the store. However, there are not differences between groups respect to affective internal state based on **Table 4** (*p*-value = 0.932).

On the other hand, we can affirm that that music is a non-verbal stimulus which does not cause differences between user’s behavioral responses, and neither considering mediating variables.

### Effects of Non-verbal Communication Stimulus (Hedonic Dimension) on User Responses: Presentation of Product

The results obtained in satisfaction construct analyzed (**Table 4**) show that consumers who were exposed to an online shopping environment with the “moving images” web designs are more satisfied those who were exposed to the “static images” conditions within the store. Moreover, this non-verbal stimulus influence positively on believes about online apparel purchase (**Table 8**).

Regarding the effects of non-verbal communication stimulus on behavioral responses (i.e., approach responses and real shopping outcomes), MANOVA test shows significant differences (**Table 5**). The ANOVA test (**Table 8**) indicates that users exposed to “moving images” stimulus show more favorable behavior intention and opinion about the website that the users exposed to “static images.” Moreover, all real shopping outcomes (i.e., products bought, time spent during the visit and money spent) are more positives in a “moving images” web design. Specifically, the involvement (with apparel and with online shopping) and perceived risk affect positively on approach responses. In contrast, only involvement with apparel affect positively on real shopping outcomes. The rest of mediated factors considered have not significant influence on behavioral responses.

## Discussion, Limitations and Future Research

The S-O-R paradigm can be useful to illustrate the influence of web atmosphere on consumers. This model indicates that external stimuli (like web atmospheric cues) affect consumers’ internal states and, in turn, they have an effect on behavioral responses, within an online shopping context (Mehrabian and Russell, 1974; Eroglu et al., 2001). In this work, we obtained that three dimensions manipulated (i.e., navigational structure as verbal stimulus, and music and presentation of products as non-verbal stimuli) affect significantly on internal and behavioral user responses. The results are relevant for retail marketers because they must offer attractive online store as unique or complementary sale channel to entice people into their shops.

The main contribution of this study, compared to the previous one, is that we have considered many and different variables and, what it can be more important, the inclusion of some mediator variables that can modify the consumer behavior and can explain the modification of some established relationships studied previously. Based on results, and in line with the results obtained by Dailey (2004), restrictive navigation act as barriers over web navigation. In this sense, a hierarchical navigation can be perceived as more restrictive. In addition, consumer’s personal characteristics (i.e., involvement, atmospheric responsiveness, and perceived risk) mediate these results. In general, navigational design, as utilitarian or verbal atmospheric cue, has a greater effect especially on cognitive states (i.e., knowledge, learning and beliefs on the Internet). It would be necessary and interesting to analyze the impact of internal variables as mediator factor between stimulus and behavioral responses (Lorenzo et al., 2007).

Moreover, positive user internal states (specifically, satisfaction) are more favorable with a “music” and “moving images” web designs and the behavioral responses are more probable with, specially, “static images” conditions. In addition, users’ personal characteristics also modifies relatively the final shopping human behavior.

It is necessary to take into account the inclusion of and appropriate music for target market (i.e., more dynamic for young people and more paused for old people) in order to offer users a more attractive virtual shop window. Nevertheless, according our results, the music is a web stimulus not very relevant for the sample.

Moreover, the use of verbal communication stimulus, is also important to improve the user internal states, as can be the satisfaction toward the use of a website, as Shankar et al. (2003) postulated. However, it is necessary to take into account the non-verbal stimuli, as the music or the way of presenting the products, affect directly on behavioral responses, as satisfaction. In consequence, website designers should include full products catalog, location information, searching engine and direct access to home page, in all websites of site, improves the navigation developed by user and, in turn, increase his/her internal states and shopping responses.

Other recommendation for e-marketers is to analyze the click-through. They represent real shopping outcomes such as time spent during the visit, products bought, and money spent. Web analytic allows studying the objective shopping outcomes through some free web applications like Google Analytics. The combination of subjective (i.e., internal states, approach responses, mediator variables) and objective data (i.e., click-through) offer e-marketer full information on their customers in order to analyze their habits and behaviors.

As main limitations, this research has been only focused on the analysis of relationships between three specific online atmospheric cues and internal states/behavioral responses. It is a limitation because; the internal states mediated this relationship, aspect not tested in this research. On the other hand, according to literature, these kinds of variables mediate the relationship between webtmospheric cues and consumers’ internal states (Choudhary, 2016). However, in this research their influence on internal and behavioral responses was studied in order to analyze the degree of significance in all the constructs. Moreover, the sample was composed of young people who were familiar with web media (i.e., different web designs, a lot of kinds of online stores and experience with digital products, etc.). So, perhaps perceived risk mediator variable is not significance on the satisfaction construct, due to sample is familiarity with online shopping.

Changes between treatments are not entirely *ceteris paribus*, however, there are many design conditions that change, especially in the navigation structure. This is an unavoidable problem in online experiments. So, the obtained conclusions should be stated carefully. This manuscript does not show the mixture of three manipulations (e.g., free navigation with music and dynamic graphic). It shows that the particular free navigation chosen in this experiment is better than the particular hierarchical navigation, and that the particular music played is preferred than no music, etc.

As future research, it would be interesting to analyze the user experience toward other manipulations on webmospheric (e.g., color, different navigational patterns, manipulations about marketing mix elements such as prices, promotions, as suggested Lallemand et al., 2015), and the typology of products bought, main sections visited by users, etc. Moreover, our model could be improved using a structural equation modeling in order to analyze the user’s internal variables [e.g., trust and familiarity, based on TAM (Kim, 2012) as mediator constructs between webmosphere stimuli and behavioral responses (Chen and Teng, 2013)]. Finally, it would be interesting to compare this results with other different cultures (McMahan et al., 2014) where the use of the online shopping is different, as well as other characteristics that could affect to the online shopping (e.g., extroversion, familiarity, etc.), as Hofstede et al. (2010) proposed.

Moreover, this research opens a line of research in cognitive neuroscience. According to Gazzaniga et al. (2002), cognitive neuroscience is a branch of both psychology and neuroscience, overlapping with disciplines such as physiological psychology, cognitive psychology, and neuropsychology. Therefore, to complement the results obtained from a psychological perspective about consumer behavior and perceptions, it would be necessary to develop some neuromarketing techniques to understand in a more detailed way how the consumers think and decide, which involves brain processes that our minds are not aware of. When experimental designs are combined with neuromarketing techniques, they provide insights into consumer decisions and actions that are invisible to traditional market research methodologies (Javor et al., 2013; Gani et al., 2015).

In addition, it would be necessary to study empirically the use of e-commerce by this kind of companies (Solaymani et al., 2012) in order to compare both perspectives, i.e., use of e-commerce by companies attending to webmosphere and opinion of consumers based on webmosphere stimuli received through online store.

## Ethics Statement

The study was exempt from ethics approval in accordance with the policies of the University of Castilla-La Mancha.

## Author Contributions

All authors listed have made substantial, direct and intellectual contribution to the work, and approved it for publication.

## Conflict of Interest Statement

The authors declare that the research was conducted in the absence of any commercial or financial relationships that could be construed as a potential conflict of interest.
